# Review by V. H. Talliaferro, M. D. of “The Anatomy and Physiology of the Placenta; the Connection of the Nervous Centres of Animal and Organic Life”

**Published:** 1860-12

**Authors:** John O’Reilly


### ARTICLE IV.

*Review, by* V. H. TALLIAFERRO, M.D., *of* "*The Anatomy and Physiology of the Placenta; The Connection of the Nervous Centres of Animal and Organic Life.*" By JOHN O'REILLY, M. D.

The above is the title of a most *astonishing* and *confounding* work. The author very appropriately remarks in the preface, "No person is more firmly impressed with the conviction that it requires a man of *profundity* of thought, *depth* of penetration, *expansive* intellect, and *enlarged* views, &c.," to illustrate the subject under discussion; and at the same time seems fully alive to the fact, "that no person is more forcibly convinced of his incompetency to give a subject of such extraordinary importance, ample justice" than himself. Following such an avowal, the author asks the question that would very naturally arise in every man's mind. Says he, "What, therefore, has induced me to attempt writing on a subject, which, during a period of twenty years, whilst actively engaged in the practice of Medicine and Surgery, *I never bestowed a thought on.*" Can the most reflecting and astute solve so *strange* a problem? The reply is, that while in an accidental attendance at one of the meetings of the Academy of Medicine, the author casually advanced views, which he subsequently found necessary to vindicate, and which he feels now anxious to place before the profession *in extenso.*

The above explanation reminds us somewhat of a very *imaginative* individual who "accidentally" happened along and "casually" remarked that his horse was *sixteen* feet high, when a friend suggested that he meant to say *hands* instead of *feet.* No, said he, he had said *feet*, and he would *stick to it* 'till he died.'

In the course of his introductory, the Doctor seems clearly to have forgotten his acknowledgment of incompetency, and concludes that "the great obstacle to a thorough understanding of the nervous system of animal or organic life, presents itself in the want of human intelligence of a sufficiently high standard to comprehend the agency of immaterialism in the operations of materiality." *His* intellect seems to have arrived at a sufficiently high standard for such comprehension; for, says he, "The union, therefore, of an immaterial agent, with materiality, scientifically arranged, as is exemplified in man, constitutes life," and that this agent, known as life, "is held in existence by the union of the oxygen of the blood with the structure of the organic nervous system." We are not told by what process, or series of experiments this *fact*(?) was arrived at, but suppose by some unknown *chemical* test, "scientifically arranged," by one endowed with great *profundity* of thought, *depth* of penetration, *expansive* intellect, and *enlarged* views.

Our author further proceeds to unfold the mysteries which for, lo these many ages, have so bewildered and beclouded the minds of physicians and metaphysicians:

"Whatever, therefore, disturbs this process," [*the union of oxygen with the organic nervous system, scientifically arranged of course*] "is followed by fatal consequences; as for instance, a violent blow on the semilunar, the superior cervical, the cardiac, or the central cerebral ganglia; the shock given to one ganglion, is instantly communicated to all the others; thereby the animal machinery suffers concussion, and is unable to attract or combine with the oxygen from the blood: thus the immaterial agent, whose existence depends on the harmonious action of these material agents (to wit, the organic nervous system [and the oxygen) disappears from its abode." "A dead man, and one in a sound sleep clearly resemble each other as regards communication with the external world," the difference being "in the operations of the immaterial agent in the organic nervous system being carried on in the latter, whilst in the former they are not;" that one having *life*, and the other having *no life*. We cannot conceive that great "profundity of thought" is requisite to a development of the fact, that the difference between a dead man and one who is asleep, is that the sleeping man has life, and the dead man has not, he having had the "scientific arrangement" of his oxygin and nervous system disturbed, and consequently his immaterial agent (*life*) having forsaken its abode. Is this not a subject for the *grave* consideration of metaphysicians, doctors, sages, and philosophers?

It is well that the author submits the question, whether he has made *a discovery* or not.

The writer argues, that the gray and white substances of the brain act upon the same principle that exists when two large sheets of zinc and copper are brought together for the production of electricity; the action of which is developed by an acid mixture, while the two substances of the brain are made to act one upon the other, through the agency of phosphorus. This explanation is thought to be an hypothesis, but appears to be borne out, to a certain extent, by facts, because when a man overworks his brain, phosphorus is found in the urine. We can very cordially endorse the author's suggestion, that this is an hypothesis, and think that many suggestions of like character in the course of the work would have been *apropos*.

We pass now to a brief consideration of the anatomy and physiology of the placenta. We shall, however, according to the division of the author, allude again to the *nervous system*, *oxygen*, *immateriality*, etc., etc.

Twelve pages of this very neat and handsomely bound volume are devoted to the above subject. The author takes grounds against the long established principles of endosmosis and exosmosis, for the fœtal sustenance and development. He says: "The idea that the fœtal blood receives oxygen by the process of endosmosis and exosmosis from material arterial blood, presupposes that the latter is in a condition to afford the requisite quantity of the vivifying agent, and that the uterine arteries, instead of terminating in capillaries, and thus containing, necessarily, blood of a venous character, present themselves in the shape of small fountains all over the structure of the placenta, separated by membranous partitions from the depots of fœtal blood in the fœtal tufts; in plain language, the reservoirs of the umbilical vein."

"Taking for granted this ingenious mechanical arrangement to be the correct one, it may be asked, what becomes of the carbon displaced by the oxygen? The maternal blood cannot be giving oxygen and receiving carbon at the same time. In other words, the oxygen from the maternal blood would *unite* with the carbon from the fœtal. Carbonic acid would be formed. The question now suggests itself, what becomes of the carbonic acid, as there is no channel by which it can make its escape. There is no organization resembling the bronchial tubes, or tracheæ to carry it off."

"I now submit that the placenta is composed of lobules, which William and John Hunter uses as cell or interstices; that these lobules are placed there for a specific purpose; that each lobule is composed of an uterine artery, uterine vein, hypogastric artery, and umbilical vein; that the change in the blood takes place in the lobules; that the uterine and hypogastric arteries inosculate in the lobule; that the carbonaceous matter is removed from the blood, and finds its way into the uterine veins, whilst the umbilical capillaries convey the *pure* blood to the fœtus."

Prof. Dalton, who the author says confirms his opinions, says: "There is, however, no direct communication between the fœtal and maternal vessels. The blood of the fœtus is always separated from the blood of the mother by a membrane which has resulted from the successive union and fusion of four different membranes, viz.: first, the membrane of the fœtal villus; secondly, that of the uterine follicle; thirdly, the wall of the fœtal bloodvessels; and fourthly, the wall of the uterine sinuses."

"These tufts (fœtal tufts), accordingly, in which the blood of the fœtus circulates, are bathed everywhere in the placental sinuses with the blood of the mother, and the processes of endosmosis and exosmosis, of exhalation and absorption, go on between the two with the greatest possible activity."

These opinions of Prof. Dalton are, at present, the established and received opinions of standard authorities, both of America and Europe; and if the author can make them confirmative of his own, then he has advanced *as new*, nothing but established *facts*. When the author maintains "that there must be a *vascular communication* between the respective organs" (uterus and placenta), he but maintains a theory which dates as far back as the days of the immortal Galen, and one which has long since been extinguished by the ever-growing lights of science. Galen had no *accumulated facts* from the scalpel and the microscope to guide him in the sunlight of truth, and therefore is irreproachable for false physiological teachings.

The existence of uterine sinuses, *which the author posi-*

*tively denies*, has been *establised* by repeated experiment—more recently by Prof. Dalton in the presence of a number of distinguished medical gentlemen. The writer asks the question, if the theory of endosmosis and exosmosis be the correct one, what becomes of the carbonic acid formed by the union of oxygen and carbon in the placenta, there being no bronchial tubes to carry it off? We would answer by asking this question: Can corbonic acid be conveyed by *nothing* but a bronchial tube or a treachea? Assuredly it is one of the great functions of the blood to convey carbonic acid and other excrementicious material, (the result of elements which have performed their functions, and of no further use for the development of living tissues,) from the system.

The placenta, during intra uterine life, has the same relationship to the fœtus that the lungs and intestines do after birth—the placental tufts, which are perpetually bathed in the maternal blood afforded by the sinuses, giving off excrementicious matter, while nutrient materials are being absorbed, thus affording a most striking example of the beautiful physiological principles—endosmosis and exosmosis.

Our author next takes up "THE CONNECTION OF THE NERVOUS CENTERS OF ANIMAL AND ORGANIC LIFE," in which it is claimed that the functions of organic life are not disturbed, so long as the *pineal gland* is unmolested, and gives as an example, hydrocephalus, in which the brain becomes enlarged, and the cranium expanded, the pineal gland floating uncompressed in the serum which fills the ventricles.

Again: a child is attacked with meningitis; the result is effusion of serum in the ventricles; the cranium being insufficiently large, and unable to accommodate itself upon so short notice to its increased contents, "and hence the phenomena and death of the patient; the vital principles being compressed out of the ganglion."

Such doctrines would seem to preclude the possibility of pressure or injury upon the surface of the brain being followed by serious and fatal sequences, but our author, with seeming satisfaction to himself, thus gets over the difficulty: "When a person suffers from compression of the brain, caused by bone, blood or matter, on the right side of the brain, he may recover with hemiplegia of the left side. This complication is most assuredly puzzling. How does it occur? If a man is impelled with force from behind, and strikes his breast against a resisting substance, his breast suffers and not his back. If a cushion is placed on the right arm, and then firmly pressed against, whilst the left arm is placed against a resisting body, then it is the left arm that suffers from the pressure, and not the right; precisely on the same principle when pressure is applied to the right side of the ganglion, the force falls on the left, as it comes in contact with the point of resistance. The left side of the central ganglion is thus rendered unable to communicate with the brain, spinal cord, or intervertebral ganglia at the roots of the motor nerves; hence the hemplegia."

Many such arguments are advanced to establish the *pineal gland* "as the president of the organic nervous system," in all respects as lucid and convincing as the above. Indeed, it would appear, from the writer's reasoning, that he would have the whole brain mass as but a protecting wall for the pineal gland, for he affirms that the brain is not a vital organ.

The author concludes this subject under the head of

"FURTHER *Remarks upon the Connection of the nervous Centers of Animal and Organic Life*," but before doing so, says: "I will leave it to others to decide whether I have made a discovery or not, with respect to the anatomy and physiology of the placenta, or the connection of the nervous centers of animal and organic life."

